# Association between diabetes status and breast cancer in US adults: findings from the US National Health and Nutrition Examination Survey

**DOI:** 10.3389/fendo.2023.1059303

**Published:** 2023-06-21

**Authors:** Xingyu Sun, Qiangsong Zhang, Kaisaierjiang Kadier, Pengcheng Hu, Xiaozhu Liu, Jialing Liu, Yulu Yan, Chenyu Sun, Vicky Yau, Scott Lowe, Muzi Meng, Ziru Liu, Meirong Zhou

**Affiliations:** ^1^ Department of General Surgery, The Second Xiangya Hospital, Central South University, Changsha, China; ^2^ Department of Gynecology, The Affiliated Traditional Chinese Medicine Hospital of Southwest Medical University, Luzhou, Sichuan, China; ^3^ Emergency Department, East China Hospital affiliated to Fudan University, Shanghai, China; ^4^ Department of Cardiology, First Affiliated Hospital of Xinjiang Medical University, Ürümqi, China; ^5^ Department of Ophthalmology, The Second Affiliated Hospital of Chongqing Medical University, Chongqing, China; ^6^ Department of Cardiology, The Second Affiliated Hospital of Chongqing Medical University, Chongqing, China; ^7^ Department of Phase I Clinical Trial Center, The Second Affiliated Hospital of Chongqing Medical University, Chongqing, China; ^8^ Clinical Medical School, the Southwest Medical University, Luzhou, Sichuan, China; ^9^ Department of General Surgery, the Second Affiliated Hospital of Anhui Medical University, Hefei, China; ^10^ Department of Thyroid and Breast Surgery, the Second Affiliated Hospital of Anhui Medical University, Hefei, China; ^11^ Division of Oral and Maxillofacial Surgery, Columbia University Irving Medical Center, New York, NY, United States; ^12^ College of Osteopathic Medicine, Kansas City University, Kansas City, MO, United States; ^13^ UK Program Site, American University of the Caribbean School of Medicine, Preston, United Kingdom; ^14^ Bronxcare Health System, The Bronx, NY, United States

**Keywords:** diabetes status, prediabetes, type 2 diabetes, obesity, breast cancer, NHANES

## Abstract

**Objectives:**

The aim of this study was to investigate the association between diabetes status and the risk of breast cancer among adult Americans, exploring the impact of BMI, age, and race on this relationship.

**Methods:**

A cross-sectional analysis of 8,249 individuals from the National Health and Nutrition Examination Survey (NHANES) was conducted. Diabetes was categorized as type 2 diabetes and prediabetes, with both conditions diagnosed according to the ADA 2014 guidelines. The association between diabetes status and breast cancer risk was explored using multiple logistic regression analysis.

**Results:**

Patients with diabetes had higher odds of breast cancer (OR: 1.51; 95% CI 1.00 to 2.28), Using the two-piecewise linear regression model, it was observed that there is a threshold effect in the risk of breast cancer occurrence at the age of 52 years. Specifically, the risk of breast cancer is relatively low before the age of 52 but increases significantly after this age.

**Conclusions:**

This study identified a significant association between diabetes status and breast cancer risk among adult Americans. We also found a threshold effect in breast cancer occurrence at the age of 52. Age was significantly associated with breast cancer risk in both Non-Hispanic White and Non-Hispanic Black individuals. These findings underscore the importance of diabetes management, maintaining a healthy BMI, and age-related risk considerations in reducing breast cancer risk.

## Introduction

There are more than 40 million cases of breast cancer in women worldwide and it is the second most common cancer among women in the United States (1, 2). The American Cancer Society indicates that approximately 42,000 women will die from breast cancer in 2020, with 276,000 newly diagnosed cases (3). Breast cancer affects women of all ages. However, the incidence of breast cancer increases with age, with a peak incidence at 45-64 years (4). There are many factors associated with the risk of breast cancer (5, 6). The prevalence of diabetes is increasing at an alarming rate and has become one of the most serious public health problems in the world. Diabetes is also considered to be the most common endocrine disease. The American Diabetes Association (ADA) shows that diabetes is the fourth leading cause of death in the United States (7).

There is a growing recognition that type 2 diabetes mellitus (T2DM) and breast cancer (BC) occur together in the same patient population with high mortality rates (8). Overall survival and disease-specific survival are significantly worse in diabetic BC patients compared to non-diabetic BC patients, suggesting a correlation between T2DM and cancer progression (9). Hardefeldt et al. showed that diabetes mellitus is an independent risk factor for breast cancer (10). According to the results of a meta-analysis, women with diabetes had a 23% higher risk of future breast cancer than women without diabetes (11). A meta-analysis showed that women with diabetes had a significantly higher risk (~20%) of breast cancer than those without diabetes (12). T2DM and hyperinsulinemia were independently associated with postmenopausal breast cancer (13). In addition, a growing body of data suggests that diabetes and its complications adversely affect cancer treatment (14) and increase mortality (15), thereby affecting the prognosis of breast cancer patients (16, 17). Studies have suggested that the higher risk of breast cancer among the diabetes patients can be resulted from detection bias or potential confounders (18, 19); and that the use of antidiabetic drugs might affect the risk of breast cancer.

Patients with prediabetes have higher than normal blood glucose levels, but not high enough to be considered asT2DM. However, this is often seen as a warning sign. Prediabetes is characterized by impaired fasting glucose (IFG), impaired glucose tolerance (IGT), or an HbA1c of 39 mmol/mol (5.7%) to 46 mmol/mol (6.4%) (20). The significance of prediabetes lies in the risk associated with progression to T2DM, which is disproportionately higher at the upper end of the prediabetes range and in the combined presence of impaired fasting glucose (IFG) and impaired glucose tolerance (IGT) (20). Prediabetes and T2DM are parts of a continuum of spectrum that share pathophysiology and are associated with typical phenotypes including obesity, hypertension (HTN) and dyslipidemia (DLP) (21). Despite extensive research on the association between diabetes and breast cancer, many aspects of the relationship and underlying mechanisms remain unclear. Therefore, further research in this area is necessary.The aim of this study was to investigate the relationship between diabetes status and breast cancer in United States adults using data from the National Health and Nutrition Examination Survey (NHANES) from 2011-2016. Specifically, the objectives of this study were to: 1) examine the distribution of diabetes status (T2DM, prediabetes, and non-diabetes) in the study population; 2) determine the correlation between diabetes status and breast cancer; 3) determine the relationship between race and breast cancer; and 4) determine the relationship between BMI and breast cancer. By analyzing these factors, we aimed to gain a better understanding of the risk factors associated with breast cancer in relation to diabetes status.

## Materials and methods

### Data source

NHANES is a cross-sectional, population-based survey that assesses the health and nutritional status of the United States civilian, noninstitutional population through interviews, physical examinations, and laboratory tests. It is publicly available, and data is released every two years on a nationally representative sample using a multistage probability sampling design and weights (22). The NHANES program is reviewed annually by the National Center for Health Statistics Ethics Review Committee to ensure its ethical and scientific standards (23).

### Study population

**Figure 1:**
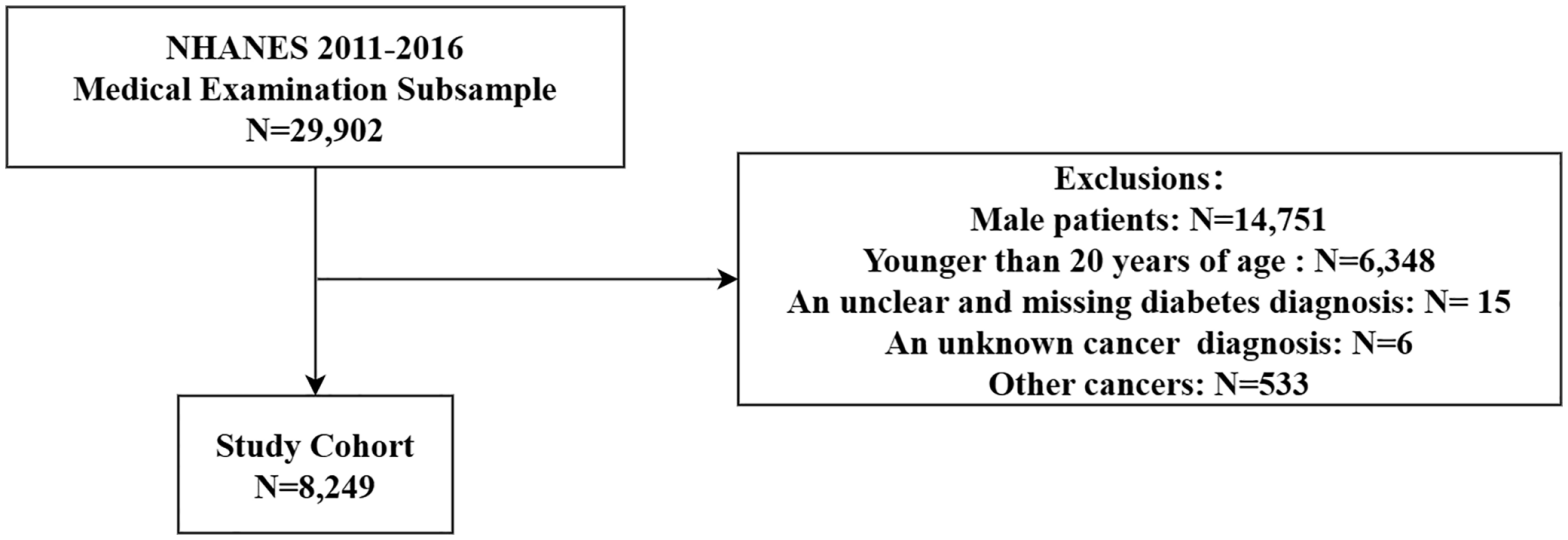
Research flowchart.

The data used in this study were obtained from the 2011-2016 survey cycle (24). This provides information on all the variables that have been used to determine the risk factors and determinants of type 2 diabetes in recent years. The process for study selection is shown in the flow diagram in **Figure 1**. Multiple interpolation was used for missing data.

### Diagnostic criteria for diabetes and prediabetes

The diagnostic criteria for type 2 diabetes mellitus (T2DM) and prediabetes are shown in **Supplement Table 1** and the study population had to meet the diagnostic criteria or have a clear diagnosis of diabetes in NHANES.

### Statistical analysis

Data were presented as weighted mean ± standard error (SE) for continuous variables and weighted percentages (95% confidence interval) for categorical variables. The associations between diabetes status and breast cancer, as well as race and breast cancer, were examined using logistic regression models. Three models were employed for the analysis: Model 1 as the crude model with no adjustments, Model 2 adjusted for age, race, and body mass index (BMI), and Model 3 adjusted for age, race, BMI, educational level, serum creatinine, cholesterol, triglycerides, glycohemoglobin, serum cotinine, estradiol, marital status, serum glucose, and reproductive health. The threshold effect analysis of BMI and age on breast cancer was assessed using two-piecewise linear regression models. The inflection points for BMI and age were determined, and odds ratios (ORs) with 95% confidence intervals (CIs) were calculated for both below and above these inflection points. The log-likelihood ratio was also reported to evaluate the goodness-of-fit of the models. Additionally, the threshold effect analysis of BMI and age for different racial groups was performed using the standard linear model, and the adjusted ORs with 95% CIs were reported for each racial group. The statistical software package R (http://www.R-project.org) was used for statistical analyses. Statistical significance was considered when the P value was < 0. 05.

## Results

### Characteristics of the participants

**Table 1** presents the weighted characteristics of the study sample, which consisted of 8,249 participants classified by NHANES and grouped by diabetes status (Type 2 diabetes, prediabetes, and non-diabetes). Our findings showed that the Type 2 diabetes group had a significantly higher BMI (33.934 kg/m2) than both the Prediabetes group (30.988 kg/m2) and Non-diabetes group (27.660 kg/m2) (p < 0.0001). Additionally, there was no significant difference in serum nicotine levels between the Type 2 diabetes group and the Non-diabetes group (p = 0.625). However, the estradiol level in the Type 2 diabetes group was significantly lower (35.884 pg/mL) than the Prediabetes group (69.905 pg/mL) and Non-diabetes group (142.538 pg/mL) (p < 0.0001). Furthermore, the age of menarche in the Type 2 diabetes group (12.534 years) was significantly lower than the Prediabetes group (12.751 years) and Non-diabetes group (12.774 years) (p < 0.001). Lastly, the age of menopause in the Type 2 diabetes group (42.751 years) was significantly higher than the Prediabetes group (41.533 years) and Non-diabetes group (35.999 years) (p < 0.0001). More detailed results can be found in **Table 1**.

**Table 1:** Weighted characteristic of study sample.

	Type 2 diabetes	Prediabetes	Non-diabetes	P-value
N	1452	2105	4692	
Body mass index (kg/m^2^)	33.934 (0.288)	30.988 (0.264)	27.660 (0.150)	< 0.0001
Cholesterol (mmol/L)	4.961 (0.041)	5.281 (0.033)	4.998 (0.019)	< 0.0001
Creatinine (umol/L)	78.413 (1.720)	69.420 (0.971)	66.153 (0.374)	< 0.0001
Serum glucose (mmol/L)	7.999 (0.124)	5.452 (0.022)	4.923 (0.015)	< 0.0001
Triglycerides (mmol/L)	2.110 (0.079)	1.611 (0.031)	1.350 (0.021)	< 0.0001
Serum Cotinine (ng/mL)	38.499 (3.590)	42.957 (3.337)	39.828 (2.471)	0.625
Estradiol (pg/mL)	35.884 (2.278)	69.905 (9.925)	142.538 (8.822)	< 0.0001
Glycohemoglobin (%)	7.049 (0.055)	5.686 (0.011)	5.242 (0.008)	< 0.0001
Age when first menstrual period occurred	12.534 (0.055)	12.751 (0.057)	12.774 (0.027)	< 0.001
Age at last menstrual period	42.751 (0.262)	41.533 (0.219)	35.999 (0.267)	< 0.0001
Age (years)				< 0.0001
≤45	257 (19.240)	638 (30.515)	2959 (62.474)	
>45	1195 (80.760)	1467 (69.485)	1733 (37.526)	
**Race**				
Non-Hispanic White	400 (55.204)	727 (64.216)	1759 (64.813)	< 0.0001
Non-Hispanic Black	422 (17.934)	518 (13.471)	984 (11.544)	
Non-Hispanic Asian	149 (5.894)	279 (6.160)	650 (5.967)	
Mexican American	243 (10.586)	290 (7.571)	596 (8.335)	
other	238 (10.381)	291 (8.581)	703 (9.340)	
Education level				< 0.0001
Less than 9th grade	256 (10.735)	237 (5.944)	311 (3.777)	
9-11th grade	231 (13.030)	264 (10.662)	514 (7.943)	
High school graduate/GED or equivalent	342 (25.544)	434 (20.415)	934 (18.726)	
Some college or AA degree AA degree	417 (34.004)	654 (32.806)	1577 (35.067)	
College graduate or above	206 (16.687)	516 (30.173)	1356 (34.487)	
Marital status				< 0.0001
Married	636 (49.564)	1001 (52.982)	2157 (51.096)	
Widowed	293 (17.923)	273 (11.529)	304 (4.736)	
Divorced	207 (13.276)	303 (13.750)	501 (10.955)	
Separated	76 (3.592)	99 (3.269)	155 (2.519)	
Never married	181 (11.437)	284 (11.362)	1129 (21.441)	
Living with partner	59 (4.207)	145 (7.108)	446 (9.253)	
Ever been pregnant				< 0.0001
no	137 (11.212)	224 (12.186)	948 (23.179)	
yes	1315 (88.788)	1881 (87.814)	3744 (76.821)	
Breast cancer				< 0.0001
no	1370 (94.187)	2041 (96.707)	4600 (97.930)	
yes	82 (5.813)	64 (3.293)	92 (2.070)	

Continuous variables were expressed as weighted mean ± standard error (SE).

Categorical variables were expressed as weighted percentages (95% confidence interval).

### Associations between diabetes status and breast cancer

**Table 2** shows the results of multiple logistic regression analysis of the association between diabetes status (non-diabetes, prediabetes, and Type 2 diabetes) and breast cancer, with odds ratios (ORs) and 95% confidence intervals (CIs) for three different models. Model 1 does not adjust for any covariates, Model 2 adjusts for age, race, and body mass index (BMI), and Model 3 adjusts for age, race, BMI, educational level, serum creatinine, cholesterol, triglycerides, glycohemoglobin, serum cotinine, estradiol, marital status, serum glucose, and reproductive health. For the non-diabetes group, the ORs in all three models are considered the reference group. For the prediabetes group, the OR in Model 1 is 1.57 (95% CI, 1.13-2.16, P=0.006), in Model 2 is 0.92 (95% CI, 0.66-1.28, P=0.627), and in Model 3 is 0.90 (95% CI, 0.64-1.26, P=0.530). For the Type 2 diabetes group, the OR in Model 1 is 2.99 (95% CI, 2.21-4.05, P<0.0001), in Model 2 is 1.63 (95% CI, 1.18-2.26, P=0.003), and in Model 3 is 1.51 (95% CI, 1.00-2.28, P=0.049). Overall, these findings suggest that Type 2 diabetes is significantly associated with an increased risk of breast cancer, even after adjusting for multiple covariates. Results are detailed in **Table 2**.

**Table 2:** Associations between diabetes status and breast cancer.

	Model 1	Model 2	Model 3
	OR (95% CI, P)	OR (95% CI, P)	OR (95% CI, P)
Non- diabetes	Reference	Reference	Reference
Prediabetes	1.57 (1.13,2.16) P=0.006	0.92 (0.66, 1.28) P=0.627	0.90 (0.64, 1.26) P=0.530
Type 2 diabetes	2.99 (2.21,4.05) P<0.0001	1.63 (1.18, 2.26) P=0.003	1.51 (1.00, 2.28) P=0.049

Model 1: Adjust for: None.

Model 2: Age, race, body mass index were adjusted.

Model 3: Age, race, body mass index, educational level, serum creatinine, cholesterol, triglycerides, glycohemoglobin, serum cotinine, estradiol, marital status, serum glucose and reproductive health were adjusted.

### Associations between prediabetes/diabetes and breast cancer by race

**Table 3** shows the results of multiple logistic regression analysis testing the relationship between race and breast cancer. The unadjusted model (Model 1) was first examined, followed by Model 2 adjusted for age and body mass index, and finally Model 3 adjusted for additional covariates, including educational level, serum creatinine, cholesterol, triglycerides, glycohemoglobin, serum cotinine, estradiol, marital status, serum glucose, and reproductive health. For each racial group and diabetes status, the odds ratios (ORs) with 95% confidence intervals (CIs) and p-values were calculated, with the non-diabetes group as the reference. For example, Model 1 showed that among non-Hispanic White individuals, those with type 2 diabetes had an increased risk of breast cancer, with an OR of 2.92 (95% CI, 1.87-4.49, P < 0.0001). The results are shown in **Table 3**.

**Table 3:** Associations between prediabetes/diabetes and breast cancer by race.

	Model 1	Model 2	Model 3
	OR (95% CI, P)	OR (95% CI, P)	OR (95% CI, P)
Non-Hispanic White			
Non- diabetes	Reference	Reference	Reference
Prediabetes	1.35 (0.86,2.10) P=0.184	0.82 (0.51, 1.29) P=0.401	0.82 (0.50, 1.30) P=0.399
Type 2 diabetes	2.92 (1.87,4.49) P<0.0001	1.64 (1.02, 2.61) P=0.039	1.46 (0.79, 2.64) P=0.215
Non-Hispanic Black			
Non- diabetes	Reference	Reference	Reference
Prediabetes	1.92 (0.82,4.51) P=0.129	1.05 (0.44, 2.52) P=0.910	1.12 (0.45,2.81) P=0.801
Type 2 diabetes	3.71 (1.74,8.24) P<0.001	1.76 (0.79, 4.09) P=0.170	2.18 (0.78,6.23) P=0.140
Non-Hispanic Asian			
Non- diabetes	Reference	Reference	Reference
Prediabetes	1.96 (0.56, 6.56) P=0.270	0.96 (0.27,3.25) P=0.941	0.98 (0.25,3.75) P=0.971
Type 2 diabetes	6.09 (2.09,18.76) P<0.001	2.27 (0.73,7.31) P=0.155	3.29 (0.66,1.60) P=0.138
Mexican American			
Non- diabetes	Reference	Reference	Reference
Prediabetes	2.79 (0.96, 8.54) P=0.060	1.25 (0.42,3.91) P=0.682	0.94 (0.29, 3.11) P=0.915
Type 2 diabetes	3.78 (1.35,11.39) P=0.013	1.24 (0.43,3.87) P=0.693	5.30 (0.10, 2.49) P=0.433
Other			
Non- diabetes	Reference	Reference	Reference
Prediabetes	1.69 (0.69,3.97) P=0.230	0.99 (0.40, 2.39) P=0.987	0.90 (0.35, 2.23) P=0.824
Type 2 diabetes	3.07 (1.39,6.77) P=0.005	1.72 (0.74, 3.99) P=0.204	1.43 (0.46, 4.19) P=0.523

Model 1: Adjust for: None.

Model 2: Age, body mass index were adjusted.

Model 3: Age, body mass index, educational level, serum creatinine, cholesterol, triglycerides, glycohemoglobin, serum cotinine, estradiol, marital status, serum glucose and reproductive health were adjusted.

### Analysis of the effect of BMI threshold on female breast cancer using a two-part linear regression model

**Figure 2:**
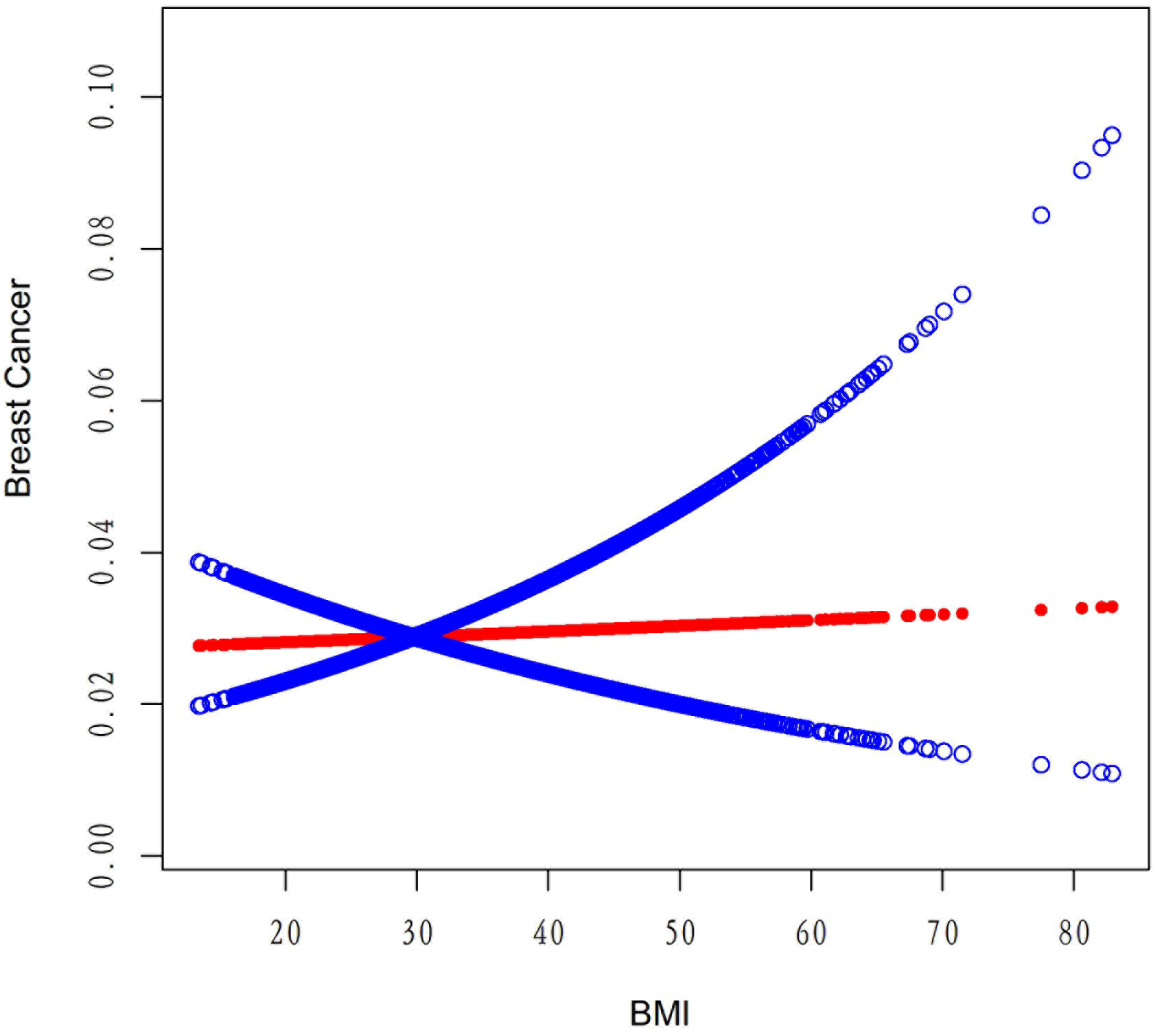
The association between BMI and breast cancer risk.

**Figure 3:**
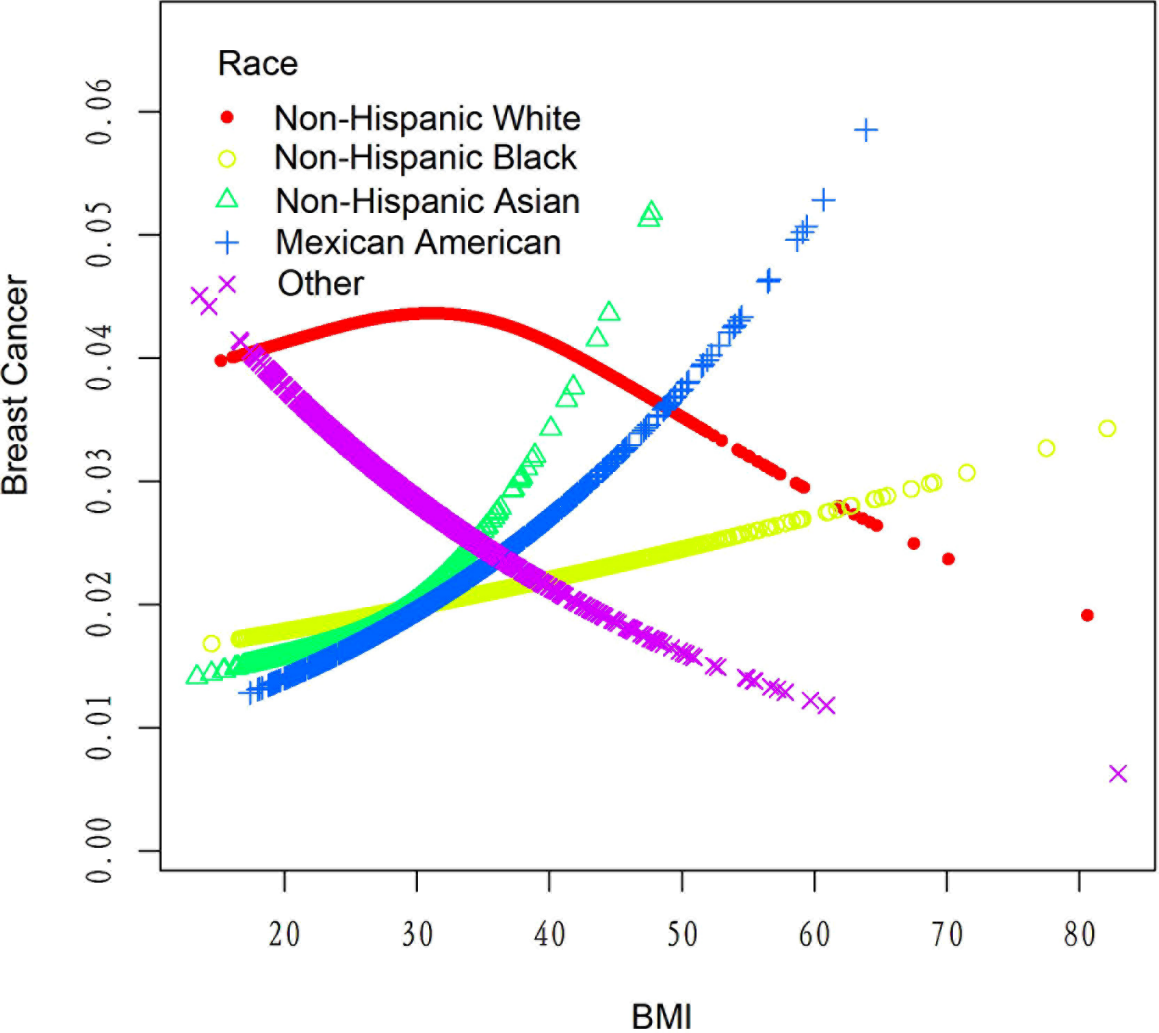
The association between BMI and breast cancer risk among different populations.

**Table 4** displays the results of a threshold effect analysis examining the relationship between body mass index (BMI) and breast cancer risk in women using a two-piecewise linear regression model. The adjusted odds ratios (ORs) with 95% confidence intervals (CIs) are presented. The table compares the results of fitting the standard linear model with those of the two-piecewise linear model. The inflection point is at 21 kg/m2. For individuals with BMI less than 21 kg/m2, the adjusted OR for breast cancer is 0.88 (95% CI: 0.69, 1.11). For individuals with BMI greater than 21 kg/m2, the adjusted OR for breast cancer is 1.01 (95% CI: 0.98, 1.03). The log-likelihood ratio is 0.297. Results are detailed in **Table 4**; **Figure 2**. **Figure 3** shows the relationship between BMI and breast cancer among different racial/ethnic groups. These findings suggest that there may be a threshold effect of BMI on breast cancer risk in women.

**Table 4:** Threshold effect analysis of BMI on breast cancer in female using the two piecewise linear regression model.

Breast cancer	Adjusted OR (95% CI)
Fitting by the standard linear model	1.00 (0.98, 1.02)
Fitting by the two-piecewise linear model	
Inflection point	21
Body mass index (kg/m^2^) < 21 (kg/m^2^)	0.88 (0.69, 1.11)
Body mass index (kg/m^2^) > 21 (kg/m^2^)	1.01 (0.98, 1.03)
Log likelihood ratio	0.297

### Threshold effect analysis of age on breast cancer in female using the two piecewise linear regression model

**Figure 4:**
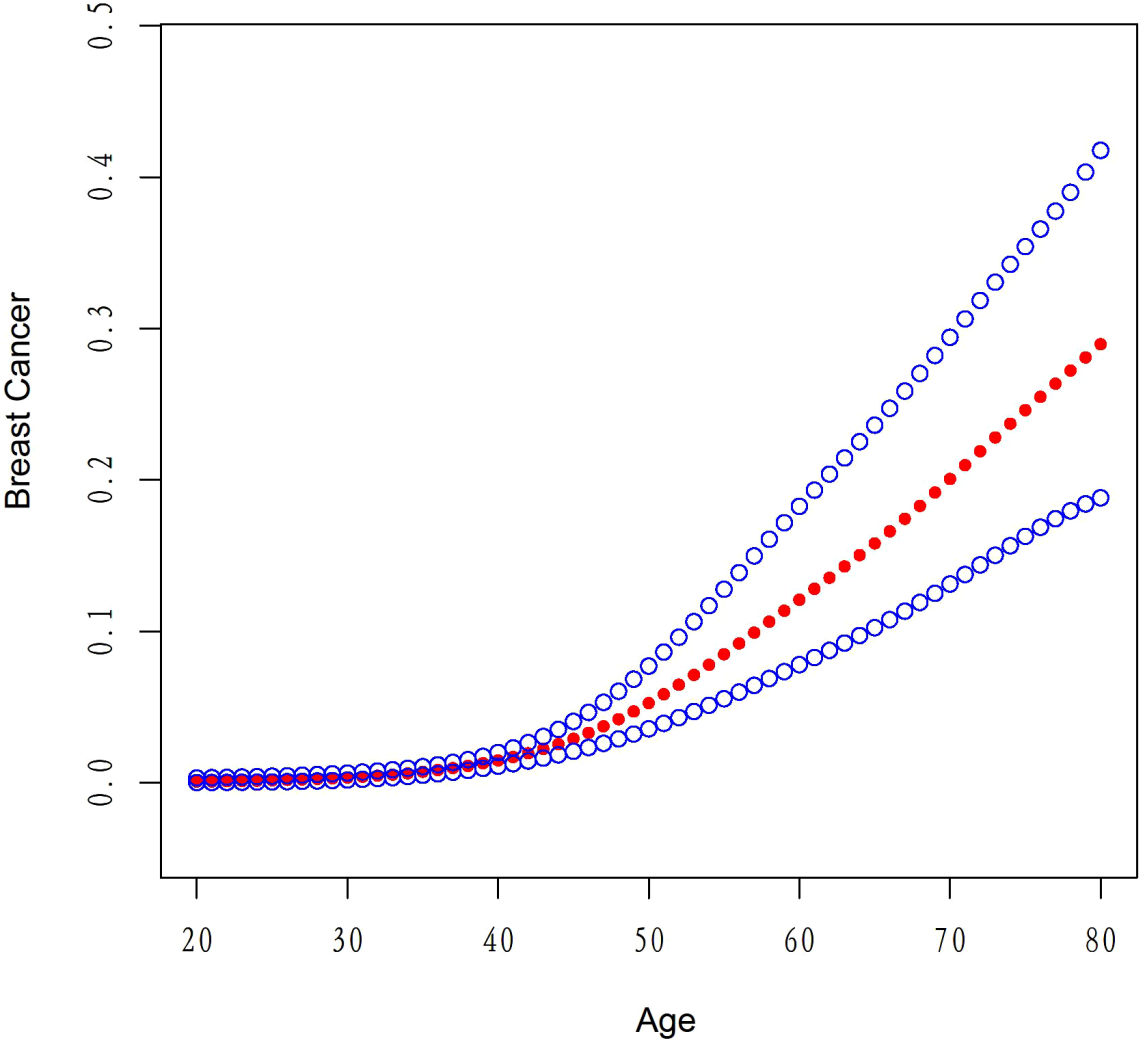
The association between age and breast cancer risk.

**Figure 5:**
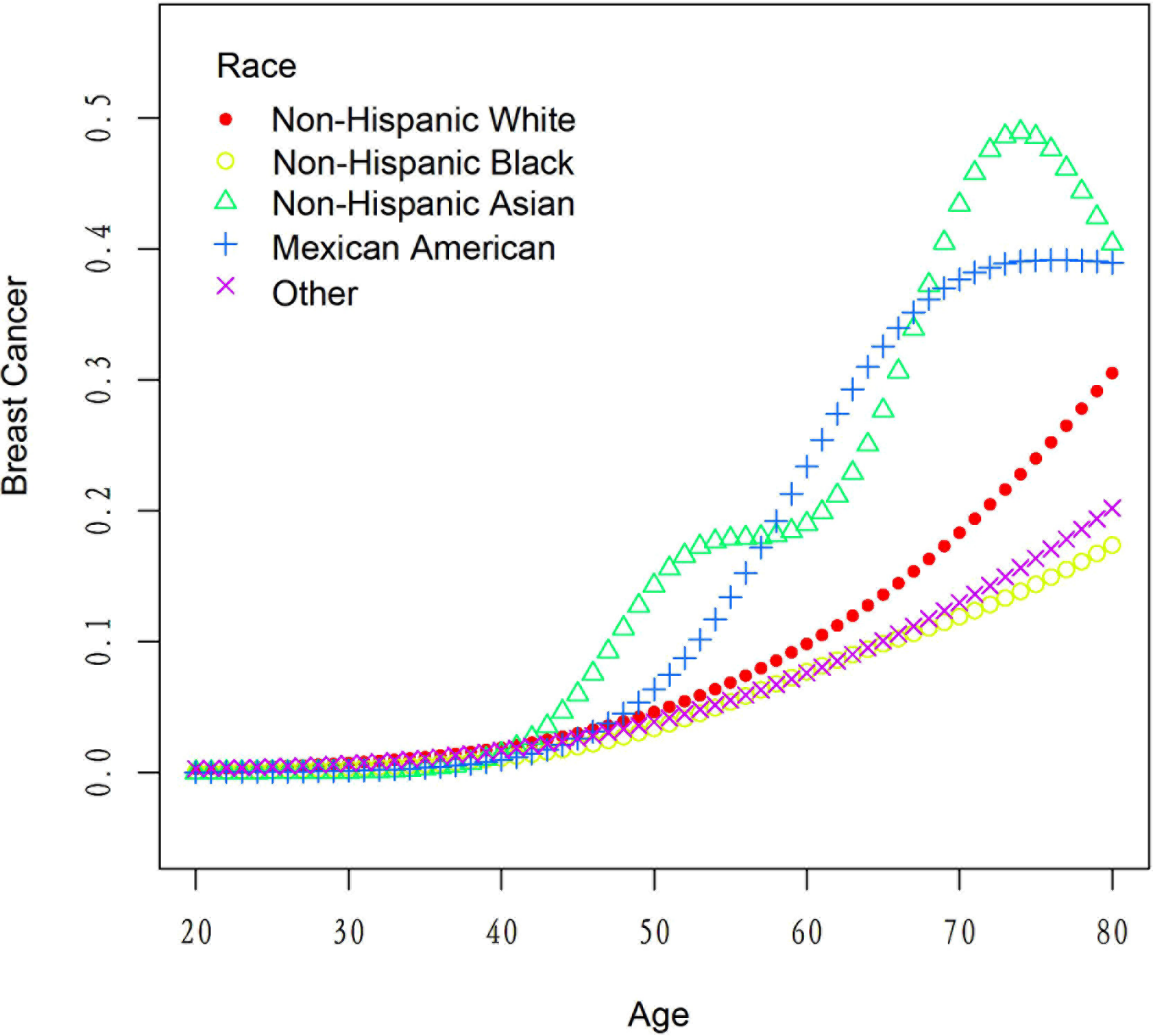
The association between age and breast cancer risk among different populations.

**Table 5** presents the results of the threshold effect analysis of age on breast cancer in females using the two-piecewise linear regression model. The table shows the adjusted odds ratios (ORs) with 95% confidence intervals (CIs) for both the standard linear model and the two-piecewise linear model. The standard linear model yielded an adjusted OR of 1.08 (95% CI: 1.06, 1.09). However, the two-piecewise linear model identified an inflection point at age 52 years. Among females aged less than 52 years, the adjusted OR was 1.18 (95% CI: 1.12, 1.26), while for those aged over 52 years, the adjusted OR was 1.06 (95% CI: 1.04, 1.08). The log-likelihood ratio was less than 0.001, indicating that the two-piecewise linear model was a better fit for the data than the standard linear model. These findings suggest that age has a threshold effect on the risk of breast cancer in females, with the risk increasing significantly after age 52 years. The results are presented in **Table 5**; **Figure 4**. **Figure 5** shows the relationship between Age and breast cancer among different racial/ethnic groups.

**Table 5:** Threshold effect analysis of age on breast cancer in female using the two piecewise linear regression model.

Breast cancer	Adjusted OR (95% CI)
Fitting by the standard linear model	1.08 (1.06, 1.09)
Fitting by the two-piecewise linear model	
Inflection point	52
age (years) < 52 (years)	1.18 (1.12, 1.26)
age (years) > 52 (years)	1.06 (1.04, 1.08)
Log likelihood ratio	<0.001

### Threshold effect analysis of BMI/Age using the standard linear model across different racial/ethnic groups

**Table 6** presents the results of the threshold effect analysis of BMI/age using the standard linear model for different racial/ethnic groups. The adjusted odds ratios (ORs) with 95% confidence intervals (CIs) and p-values are shown for each group. For Non-Hispanic White individuals, the ORs for BMI and age were 0.99 (95% CI, 0.96-1.03, P=0.7253) and 1.08 (95% CI, 1.05-1.10, P<0.0001), respectively. Similarly, for Non-Hispanic Black individuals, the ORs were 1.01 (95% CI, 0.97-1.05, P=0.6611) for BMI and 1.08 (95% CI, 1.04-1.12, P=0.0001) for age. For Non-Hispanic Asian individuals, the ORs were 1.01 (95% CI, 0.90-1.13, P=0.8865) for BMI and 1.12 (95% CI, 1.05-1.19, P=0.0005) for age. For Mexican American individuals, the ORs were 1.06 (95% CI, 0.99-1.13, P=0.1022) for BMI and 1.09 (95% CI, 1.03-1.14, P=0.0016) for age. For individuals from other racial/ethnic groups, the ORs were 0.96 (95% CI, 0.90-1.02, P=0.2252) for BMI and 1.06 (95% CI, 1.02-1.10, P=0.0031) for age. The results indicate that the association between BMI/age and breast cancer risk varies across different racial/ethnic groups. **Table 6**; **Figures 3**, **5** display the results.

**Table 6:** Threshold effect analysis of BMI/Age using the standard linear model.

	BMI		Age	
	Adjusted OR (95% CI)	P value	Adjusted OR (95% CI)	P value
Non-Hispanic White	0.99 (0.96, 1.03)	0.7253	1.08 (1.05, 1.10)	<0.0001
Non-Hispanic Black	1.01 (0.97, 1.05)	0.6611	1.08 (1.04, 1.12)	0.0001
Non-Hispanic Asian	1.01 (0.90, 1.13)	0.8865	1.12 (1.05, 1.19)	0.0005
Mexican American	1.06 (0.99, 1.13)	0.1022	1.09 (1.03, 1.14)	0.0016
other	0.96 (0.90, 1.02)	0.2252	1.06 (1.02, 1.10)	0.0031

## Discussion

The present study investigated the associations between diabetes status, BMI, age, and breast cancer risk in a representative sample of US adults, using data from the NHANES. Our analysis revealed significant relationships between diabetes status, BMI, and age with breast cancer risk, with varying associations observed across different racial groups. Our results demonstrated that individuals with Type 2 diabetes had a significantly higher risk of breast cancer compared to those without diabetes. This association persisted even after adjusting for multiple covariates, such as age, race, BMI, and other potential confounders. These findings are in line with previous research indicating that Type 2 diabetes may be associated with an increased risk of breast cancer (25). Possible explanations for this relationship include hyperinsulinemia, insulin resistance, and chronic inflammation, which have been suggested to contribute to breast cancer development and progression (26). In addition, our study showed that individuals with prediabetes had no significant increase in breast cancer risk compared to those without diabetes. This finding emphasizes the need for further research to understand the role of glycemic control and potential interventions to reduce breast cancer risk among individuals with diabetes.

Our threshold effect analysis revealed an inflection point at 21 kg/m² in the relationship between BMI and breast cancer risk. For individuals with a BMI greater than 21 kg/m², the risk of breast cancer increased, whereas those with a BMI less than 21 kg/m² had no significant change in risk. These findings are consistent with previous research demonstrating that higher BMI is associated with an increased risk of postmenopausal breast cancer (27). Several mechanisms have been proposed to explain this relationship, including increased estrogen production in adipose tissue, altered adipokine and insulin signaling, and increased inflammation (28). Our analysis also identified a threshold effect of age on breast cancer risk, with a significant increase in risk observed after the age of 52 years. This finding is in line with existing literature, which has consistently reported that breast cancer risk increases with age, particularly after menopause (29). The increased risk at older ages may be attributed to the accumulation of genetic and epigenetic changes over time, as well as age-related changes in hormone levels and immune function (30).

In our study, we discovered that the relationships between BMI, age, and breast cancer risk exhibited variations across different racial groups. However, it is important to note that the differences in the association between BMI and breast cancer risk among various racial groups were not statistically significant. This finding highlights the complexity of the relationship between BMI and breast cancer risk, and suggests that further research is necessary to better understand the underlying factors that may contribute to these variations, such as differences in body fat distribution, hormone levels, and genetic factors (31). On the other hand, age was found to be significantly associated with breast cancer risk across all racial groups, emphasizing the importance of age as a universal risk factor for breast cancer (32).

Our study has several limitations that should be considered when interpreting the findings. First, the cross-sectional nature of the data precludes establishing causal relationships between diabetes status, BMI, age, and breast cancer risk. Longitudinal studies are needed to confirm these associations and investigate potential underlying mechanisms. Second, the reliance on self-reported data may introduce recall bias, particularly for variables such as age at menarche and age at menopause. Future studies could benefit from objective measures to minimize potential biases. Third, although we adjusted for multiple covariates, residual confounding cannot be ruled out. There may be additional unmeasured factors, such as genetic predisposition, environmental exposures, and lifestyle factors, that contribute to the observed associations.

Despite these limitations, our study provides valuable insights into the relationships between diabetes status, BMI, age, and breast cancer risk in a diverse US population. Our findings highlight the importance of considering these factors in breast cancer prevention strategies and suggest that targeted interventions for individuals with Type 2 diabetes may be beneficial in reducing breast cancer risk. Moreover, our results underscore the need for further research to understand the mechanisms underlying the associations between diabetes status, BMI, age, and breast cancer risk, as well as the potential differences in these relationships across racial groups.

Future research should aim to replicate our findings in larger, prospective cohorts and investigate the biological pathways linking diabetes, obesity, and age to breast cancer development. Additionally, intervention studies targeting glycemic control, weight management, and other modifiable risk factors could help determine the effectiveness of such strategies in reducing breast cancer risk among individuals with diabetes and those with higher BMI. Finally, understanding the racial differences in the relationships between these factors and breast cancer risk may contribute to the development of more targeted and effective prevention strategies for different populations.

## Conclusion

In conclusion, our study demonstrates significant associations between diabetes status, BMI, age, and breast cancer risk in a representative US population. These findings highlight the importance of considering these factors in breast cancer prevention efforts and suggest that targeted interventions may be warranted to reduce breast cancer risk among individuals with Type 2 diabetes and those with higher BMI. Further research is needed to elucidate the underlying mechanisms and identify effective prevention strategies for diverse populations.

## Data availability statement

The original contributions presented in the study are included in the article/**Supplementary Material**. Further inquiries can be directed to the corresponding author.

## Ethics statement

Ethical review and approval was not required for the study on human participants in accordance with the local legislation and institutional requirements. Written informed consent from the patients/participants or patients/participants’ legal guardian/next of kin was not required to participate in this study in accordance with the national legislation and the institutional requirements.

## Author contributions

XS, QZ, and KK: They were responsible for drafting the work and critically revising it for important intellectual content. PH, XL, JL, YY, CS, VY, SL, and MM: They contributed to the acquisition, analysis, and interpretation of data for the work. ZL and MZ. All authors contributed to the article and approved the submitted version.
