# Supplementary material for: Association between diabetes status and breast cancer in US adults: findings from the US National Health and Nutrition Examination Survey
Source: Front Endocrinol (Lausanne). 2023 Jun 21;14:1059303. doi: 10.3389/fendo.2023.1059303 (PMC10321597; doi:10.3389/fendo.2023.1059303)
Supplement: Supplementary file 1 [file Table_1.docx]

**Supplementary Table 1: Criteria for the diagnosis of diabetes**

| Criteria for the diagnosis of diabetes: |
| --- |
| FPG≥126mg/dL（7.0mmol/L）.Fasting is defined as no caloric intake for at least8h.* |
| OR |
| 2-hPG≥200mg/dL（11.1mmol/L during OGTT.The test should be performed as described by WHO, using a glucose load containing the equivalent of 75 g anhydrous glucose dissolved in water.* |
| OR |
| A1C≥6.5%（48m mol/mol）.The test should be performed in a laboratory using a method that is NGSP certified and standardized to the DCCT assay*. |
| OR |
| In a patient with classic Symptoms of hyperglycemia or hyperglycemic crisis,a random plasma glucose ≥200mg/dl（11.1mmol/L）. |

DCCT, Diabetes Control and Complications Trial; FPG, fasting plasma glucose; OGTT, oral glucose tolerance test; WHO, World Health Organization；2-h PG，2-h plasma glucose. In the absence of unequivocal hyperglycemia, diagnosis requires two abnormal test results from the same sample or in two separate test samples.
