# Supplementary material for: Association between diabetes status and breast cancer in US adults: findings from the US National Health and Nutrition Examination Survey
Source: Front Endocrinol (Lausanne). 2023 Jun 21;14:1059303. doi: 10.3389/fendo.2023.1059303 (PMC10321597; doi:10.3389/fendo.2023.1059303)
Supplement: Supplementary file 2 [file Table_2.docx]

**Supplementary table 2 Associations between race and breast cancer.**

|  |  | Model 1 | Model 2 | Model 3 |
| --- | --- | --- | --- | --- |
|  | Case/Participants | OR (95% CI, P) | OR (95% CI, P) | OR (95% CI, P) |
| Menopause, no |  |  |  |  |
| Non- diabetes | 24/3402 | Reference | Reference | Reference |
| Prediabetes | 9/895 | 1.43(0.63,2.98)  P=0.363 | 0.94(0.40, 2.00)  P=0.869 | 1.02(0.43, 2.22)  P=0.963 |
| Type 2 diabetes | 5/479 | 1.48(0.50,3.60)  P=0.424 | 0.84(0.27, 2.19)  P=0.745 | 0.83(0.26, 2.22)  P=0.722 |
| Menopause,yes |  |  |  |  |
| Non- diabetes | 68/1290 | Reference | Reference | Reference |
| Prediabetes | 55/1210 | 0.86(0.59,1.23)  P=0.402 | 0.84(0.58, 1.21)  P=0.347 | 0.74(0.51, 1.08)  P=0.116 |
| Type 2 diabetes | 77/973 | 1.54(1.10,2.17)  P=0.012 | 1.56(1.09, 2.24)  P=0.015 | 1.41(0.95, 2.07)  P=0.086 |
|  | Model 1: adjust for: None  Model 2: age, race~~,~~ body mass index were adjusted.  Model 3: age, body mass index, educational level, serum creatinine, cholesterol, triglycerides, glycohemoglobin, serum cotinine, estradiol, marital status, serum glucose and reproductive health were adjusted. | | | |
